# Complete genome sequence of *Lachancea thermotolerans* strain L-84 isolated from Crimean vineyards

**DOI:** 10.1128/mra.01231-25

**Published:** 2025-12-22

**Authors:** Alexey V. Beletsky, Egor A. Vasyagin, Maxim Yu. Shalamitskiy, Karina A. Semenova, Nikolai V. Ravin, Andrey V. Mardanov

**Affiliations:** 1 Institute of Bioengineering, Research Center of Biotechnology of the Russian Academy of Sciences 204333 , Moscow, Russia; 2 All-Russian National Research Institute of Viticulture and Winemaking “Magarach” of the National Research Centre “Kurchatov Institute”, Yalta, Russia; University of California Riverside, Riverside, California, USA

**Keywords:** *Lachancea thermotolerans*, complete genome, winemaking, lactic acid

## Abstract

The yeast *Lachancea thermotolerans* of the family *Saccharomycetaceae* is used in winemaking to increase the acidity of wine and improve its aromatic profile. We report the complete genome sequence of *L. thermotolerans* strain L-84, isolated from Sauvignon grapes from a vineyard in Crimea.

## ANNOUNCEMENT

The *Saccharomycetaceae* yeast *Lachancea thermotolerans*, previously known as *Kluyveromyces thermotolerans* (1), is of significant biotechnological interest due to its uncommon ability to partially convert fermentable sugars into L-lactic acid instead of ethanol during alcoholic fermentation, which increases the acidity and improves the overall quality and stability of wines (2–6). Because of this trait, *L. thermotolerans* is widely used in warm-climate wine regions, where it helps to balance high alcohol levels and low natural acidity in wines. Furthermore, *L. thermotolerans* synthesizes polysaccharides, thereby enhancing the wine’s aromatic profile through the formation of esters and improving the color stability of grape must (7). *L. thermotolerans* has a broad habitat range and can be found on grapes and in other habitats, such as soil, insects, and plants (8). Genomic data from new *L. thermotolerans* isolates will reveal genetic features unique to these yeasts.

We present the complete genome sequence of *L. thermotolerans* strain L-84. The strain was isolated in 2017 from Sauvignon Blanc grapes in a vineyard located in the village of Romashkovo, near the city of Yevpatoriya, Crimea. The strain was obtained from the Collection of Winemaking Microorganisms of the Magarach All-Russian National Research Institute for Viticulture and Winemaking. The strain was grown in YPD broth (1% yeast extract, 2% peptone, 2% glucose) at 30°C for 2 days.

Genomic DNA was extracted using DNeasy PowerSoil DNA Isolation Kit (Qiagen, Germany). For Illumina sequencing, a library was constructed with the NEBNext Ultra II DNA Library Prep Kit (New England Biolabs, USA). Sequencing on an Illumina MiSeq platform (Illumina, USA) produced 1,351,728 read pairs (2 × 300 nucleotides). Adapter sequences were removed using Cutadapt v.4.0 (9), overlapping reads were merged with FLASH v.1.2.11, “-M 300” parameter was set (10). Low-quality read ends were trimmed using Sickle v.1.33 (https://github.com/najoshi/sickle) with a quality threshold of Q30. Additionally, the genome was sequenced on a MinION (Oxford Nanopore Technologies, UK). A genomic library was prepared using the Ligation Sequencing gDNA kit (SQK-LSK109) and sequenced on FLO-MIN110 flow cells generating 55,042 reads with a mean length of 4.647 nt. Nanopore reads were *de novo* assembled using Flye v2.9.5 (11) with “--nano-hq” and “--iterations 2,” consensus of the assembly was polished with Illumina reads using POLCA tool of MaSuRCA v.4.1.4 assembler (12). As a result, the complete genome of *L. thermotolerans* strain L-84 was assembled, consisting of a nuclear genome of 11,069,242 bp in size, made of 8 chromosomes, and a circular mitochondrial genome of 23,615 bp in size (Table 1).

**TABLE 1:** Genomic sequencing and assembly statistics

Parameter	Value
Illumina sequencing	
Read number	2,703,456
Total bases (nt)	813,740,256
Nanopore sequencing	
Read number	55,042
Total bases (nt)	255,755,807
Genome assembly	
Total length, bp	11,069,242
Genome coverage	73×
N50, bp	1,558,908
Chromosome A, bp	712,453
Chromosome B, bp	871,921
Chromosome C, bp	1,016,944
Chromosome D, bp	1,558,908
Chromosome E, bp	1,498,636
Chromosome F, bp	1,638,594
Chromosome G, bp	1,724,291
Chromosome H, bp	2,023,880
Mitochondrial genome, bp	23,615
GC content (%)	47.11
Protein-coding genes	6,222
tRNA genes	229
Copies of rRNA gene cluster	~74
Noncoding DNA (%)	28.61
Genome assembly assessment	
Complete BUSCOs	99.5%
Fragmented BUSCOs	0.0%
Missing BUSCOs	0.5%

GeneMark-EP v.4.71 (13) with hints from protein splice alignments was used to predict gene structures. Hints were predicted using ProtHint v.2.6.0 (13) pipeline with fungi subset of OrthoDB as a reference database. “--fungus” parameter was specified for both GeneMark-EP and ProtHint. Genome completeness was assessed using BUSCO v.5.7.1 (14) with *Saccharomycetes* database.

Taxonomic assignment based on complete genomes from the NCBI database (accessed 27 Sep 2025) showed that the closest genome belongs to *L. thermotholerans* strain CBS 6340 (GCA_000142805.1), with an average nucleotide sequence identity of 98.89%, calculated by FastANI v.1.33 (15). Default parameters were used for all software unless otherwise noted.

These genomic data will be useful for comparative genomics analysis of *L. thermotolerans* and for applying these yeasts in the food industry, including winemaking.

## Data Availability

This BioProject has been deposited in GenBank under accession number PRJNA1019646, and raw reads have been deposited in the NCBI Sequence Read Archive under the accession numbers SRR35184349 (MiSeq) and SRR35184348 (MinION). The assembled genome sequences have been deposited in GenBank under the accession number JBRIII000000000. The version described in this paper is the first version.
